# Editorial: Stress, pain or drug addiction: epigenetics, biological mechanisms and therapeutics

**DOI:** 10.3389/fnmol.2023.1298870

**Published:** 2023-10-19

**Authors:** Hee Young Kim, Insop Shim

**Affiliations:** ^1^Department of Physiology, Yonsei University College of Medicine, Seoul, Republic of Korea; ^2^Department of Physiology, College of Medicine, Kyung Hee University, Seoul, Republic of Korea

**Keywords:** stress, pain, drug addiction, epigenetics, resilience

We opened the Research Topic regarding biological mechanisms and therapeutics in stress, pain and/or addiction. Cumulating evidence highlights substantial central nervous system (CNS)-mechanism overlaps among stress, pain and addiction. Stress and pain are interacting and influencing each other at multiple levels, including genetic, biological, cognitive, behavioral, and social factors (Ziadni et al., 2022). Early and adult stressful life events modulate pain perception (Feuerstein et al., 1985) and are also risk factors for the development of addiction and serve as cues that trigger drug relapses (Sinha, 2007). Addiction is a progressive disease that affects the brain's neurology and negatively impacts on health, social, and economic welfare. Stress, pain and/or addiction are interacting or influencing each other and may have common patho-physiological mechanisms at the neural circuits and molecular targets (Elman and Borsook, 2016).

The aim of this Research Topic was to collect the current knowledge and advances in the investigation dealing with stress, pain and addiction, including resilient factors, epigenetic approaches, animal models or therapeutics. The following 5 manuscripts were published in this Research Topic after peer-review process by the experts in this field.

First, Cha et al. identified specific neural circuits underlying gender difference in pain sensitivity. Differences between male and female in brain structure and function contributing to behavior and cognition have been reported (Tyan et al., 2017). However, sex difference in pain sensitivity and pain signaling pathways has been under-explored. To explore it, Cha et al. performed pain behavior test and brain diffusion tensor imaging (DTI) tractography in male and female rats and found hypersensitivity of female rats and gender differences in pain-associated brain connectivity such as the anterior cingulate cortex (ACC), prefrontal cortex (PFC) and ventral thalamus. They suggest the existence of sexual dimorphism of brain associated with pain sensitivity (Cha et al.).

Second, Kim et al. demonstrated a critical role of hypothalamic orexinergic neurons between stress and psychiatric diseases. While it is well known that stress is an important precipitating factor for the development of psychiatric conditions for major depression (Lloyd, 1980), there are considerable variations in individual responses to the same stress, possibly due to individual variations in stress resilience (Karatsoreos and McEwen, 2013). The factors that determine stress susceptibility and resilience are poorly understood. Kim et al. investigated the role of hypothalamic orexin-expressing neurons in the regulation of stress resilience in male mice and showed that orexinergic neurons were strongly activated by acute stress and that resilient animals revealed higher activation of orexinergic neuron after chronic stress than susceptible animals. The study highlights that hypothalamic orexinergic neurons may be important for therapeutic paradigm aimed at increasing resilience in stress-associated psychiatric diseases.

Third, Jang et al. also emphasized the importance of hypothalamic orexinergic neurons in controlling addictive behaviors. The lateral hypothalamus (LH) plays an important physiological role in brain function and also plays a critical role in substance abuse. The neuropeptides called orexin (or hypocretins) have been identified as being located exclusively in the cell bodies of the LH. Kim's group has previously demonstrated that mechanical stimulation (MS) of the ulnar nerve produces strong inhibitory effects on cocaine addiction–like behaviors through an LH-lateral habenula (LHb) circuit (Ahn et al., 2021). In the present study, their findings extended that mechanical stimulation applied to the ulnar nerve activates the orexin-operated projection from the LH to the LHb and attenuates the psychomotor responses induced by cocaine. They proved that lateral hypothalamic orexinergic neurons play a pivotal role in inhibition of cocaine addictive behaviors by somatosensory stimulation (Jang et al.).

Fourth, Liao et al. endeavored to uncover a novel neural circuit overlapping stress and pain. Many patients with stress-related emotion disorders are comorbid with chronic abdominal pain, indicating that stress-primed emotional status may enhance abdominal pain magnitude (Fadgyas-Stanculete et al., 2014). To explore the limbic neuronal circuits and biochemical substrates underlying this stress-induced abdominal pain, Liao et al. investigated the mediation of a novel medial septal-lateral septal-accumbal circuit in stress-induced abdominal pain in mice. By using retrograde tracing methods, they identified a medial septal GABAergic neurons projecting to the lateral septum-accumbus which modulated stress-primed abdominal pain. In their report, activation of the circuit reduced stress-induced abdominal pain while inhibition of the circuit enhanced stress-primed pain (Liao et al.). Their research suggests that stress may cause the plastic changes of medial septal-lateral septal-accumbal circuit and thus produces stress-primed abdominal pain.

Last, Yeo and Roh provided a therapeutic mechanism of rapamycin in an animal pain model and its signaling pathway. Neuropathic pain in the orofacial area is commonly observed in humans and markedly reduces the quality of general life by disrupting food intake, face washing, and tooth brushing (Costigan et al., 2009). Yeo's colleagues previously proved that an mTOR inhibitor rapamycin suppressed orofacial formalin-induced pain (Yeo et al., 2021). Therefore, to elucidate its underlying mechanism, Yeo and Roh explored the effect of rapamycin on the orofacial pain by nerve injury and the changes of mTOR signaling-related proteins, ERK, JNK, p38 MAPK, and glial cells in the trigeminal nucleus caudalis (TNC). They demonstrated that rapamycin alleviated both mechanical and cold allodynia in orofacial pain mice. Enhanced p-p38 MAPK and p-MKK4 in orofacial pain mice were suppressed following rapamycin treatment. They also showed that the increased p-p38 expression colocalized with microglia but neither neurons nor astrocytes and suggested that suppression of orofacial pain by rapamycin is associated with the inhibition of p-MKK4/p-p38 MAPK-mediated microglial activation in the TNC (Yeo and Roh).

The above articles proved the sexual dimorphism of brain associated with pain, a novel neural circuit in stress-primed pain, the role of microglia and associated downstream signaling in pain medication, the critical role of lateral hypothalamic orexinergic neurons in addiction and increasing resilience of stress-associated psychiatric diseases. While the articles revealed the distinct and shared mechanisms between stress and pain or between stress and psychiatric diseases, no articles regarding the shared mechanism underlying pain and addiction were collected. Despite the limitation of our Research Topic, we believe that the above publications contribute to our current understanding in stress and/or pain or addiction and also help for elucidating the underlying mechanisms and discovering new therapeutics against stress, pain and/or addiction. We appreciate all authors and reviewers for their substantial contribution to this Research Topic.

## Author contributions

HK: Writing — original draft, Writing — review & editing. IS: Writing — original draft, Writing — review & editing.
